# A summary of pain and pain-related variables in the Avon Longitudinal Study of Parents and Children

**DOI:** 10.12688/wellcomeopenres.22815.1

**Published:** 2024-09-12

**Authors:** Amanda Ly, Emma Fisher, James P. Dunham, Josefin Attermo Dufva, Kate Northstone, Abbie Jordan, Anthony E. Pickering, Rachael Gooberman-Hill, Edmund Keogh, Rebecca M. Pearson, Hannah Sallis

**Affiliations:** 1 Centre for Academic Mental Health, Population Health Sciences, Bristol Medical School, University of Bristol Medical School, Bristol, England, UK; 2 MRC Integrative Epidemiology Unit, Population Health Sciences, Bristol Medical School, University of Bristol Medical School, Bristol, England, UK; 3 Department for Health, University of Bath, Bath, England, UK; 4 Anaesthesia, Pain and Critical Care Sciences, School of Physiology, Pharmacology & Neuroscience, University of Bristol, Bristol, UK; 5 Population Health Sciences, Bristol Medical School, University of Bristol Medical School, Bristol, England, UK; 6 Department of Psychology & Centre for Pain Research, University of Bath, Bath, England, UK; 7 University of Bristol Medical School, Bristol, England, UK; 8 Department of Psychology, Manchester Metropolitan University, Manchester, England, UK

**Keywords:** pain, ALSPAC, longitudinal data, impact, cohort

## Abstract

**Background:**

To study pain, data on pain characteristics, possible triggers and consequences - such as the impact of pain on people’s lives - need to be available. When not collated, described and/or organised in a systematic manner, it can be difficult to assess how useful an existing dataset may be for one’s project. This data note describes and categorises the complex and multi-modal indices of pain available in the Avon Longitudinal Study of Parents and Children (ALSPAC).

**Methods:**

Data from two generations of the ALSPAC cohort; index child participants (Generation 1, G1), their mothers and fathers/mothers’ partners (Generation 0, G0) were used. Search terms such as ‘pain’, ‘ache’, ‘hurt’, ‘sore’, specific pain conditions, labour pain and methods of pain relief were used to identify pain and pain-related variables. These data were extracted from all waves of data collection. We developed pain categories and subsequently categorised variables in an iterative process. Repeated measurements of the same variables over waves of data collection were also identified.

**Results:**

We identified 21 categories of pain variables, which were subsequently grouped into themes: pain characteristics, extended pain characteristics and causes, treatment for pain, pain interference and pain-related to specific events. Pain and pain-related data have been collected from G1 participants, G0 mothers, and G0 partners, although there are fewer data for the partners. There were some repeated measurements, most commonly, of pain location. As is typical with longitudinal birth cohort studies, maternal proxy-reports were used during participants’ younger years and self-reports were utilised from adolescence onwards.

**Conclusions:**

Researchers interested in studying pain can feasibly do so in two generations of a regional UK population who have been followed up over 30 years. ALSPAC can be used to study pain from the early years through to young adulthood and in mothers from the perinatal period onwards.

## Plain language summary for the pain data note

Researchers sometimes use existing datasets to study pain. Data may be available on different aspects of pain. For example, the intensity of pain, which part(s) of the body hurt, possible causes of pain and how pain may affect the individual’s life. These existing datasets typically come from studies relating to health and wellbeing that were set up many years ago, often from birth, and may still be active. Researchers can apply to use data from these studies to answer health-related research questions. Although these data are available, researchers may not know how useful data from existing health-related studies may be for a new project that is being planned. Looking at a data note on a specific health topic is valuable to researchers as relevant data have been collated and organised to make it easier to see what data already exists. In this data note, the authors have collated and organised data on pain from an existing study called the Avon Longitudinal Study of Parents and Children (ALSPAC). ALSPAC was set up in the early 1990s to study health and development. ALSPAC was not specifically set up to study pain. The authors found that there was a wealth of information and questions asked about pain that could be organised into 21 pain categories. The data from these questions is described in this data note and comes from the original children recruited into the study, their mothers and their fathers/mothers’ partners. The authors also found that some pain related data were collected in the same way at multiple time points (e.g. across a number of years or months) allowing researchers to look at patterns over time. Researchers interested in studying pain can use data from ALSPAC, which has data collected on two generations of a regional UK population for more than 30 years.

## Introduction

The International Association for the Study of Pain defines pain as: “an unpleasant sensory and emotional experience associated with, or resembling that associated with, actual or potential tissue damage ^1^.” Pain is experienced in some form by most people and is considered a subjective experience that varies in nature and characteristics, e.g., localised, widespread. Pain classification can also refer to temporal features, with ‘acute’ pain usually considered as transitory, whereas chronic pain is usually defined as pain that, “persists or recurs for more than three months ^2^.” The impact of pain can be considerable, affecting cognitive, physical and emotional domains as well as broader aspects of life, including social activities and financial stability ^3^.

The impact of pain can vary within and between individuals e.g., high to low impact ^4^, which in turn can intersect with temporal pain states (acute vs. chronic). The transitions between pain states (each with high to low impact), and their potential worsening or resolution are of active research interest ^4^. There are wider public health and societal motivations to identify ways to reduce the incidence and impact of pain. Whilst pain affects individuals across the lifespan, childhood and adolescence may be a critical period of pain development that may have greater weighting on later prognosis due to rapid physical and psychological growth ^5^. Prevalence estimates of paediatric chronic pain vary considerably, from 6% to 57%, with variability in the definition of chronic pain used ^6^. Reported pooled prevalence estimates of chronic pain in adults vary from 31% (pooled; 95% CI 30.8%, 31.2%) to 43.5% (pooled; 95% CI 38.4%, 48.6%) from systematic reviews and meta-analyses, with the latter being UK-specific ^7, 8^.

Researchers clearly wish to better understand pain. Self-reports or proxy-reports of key pain characteristics are often used; it may involve reporting the site of pain (i.e., which areas of the body), its sensory characteristics, its frequency and intensity, and associations with other symptoms or conditions. Pain is assessed through self-report wherever possible. Self-reports depend on subjective insight and can be prone to recall bias but given the subjective and internal experience of pain, they remain the most appropriate method. There is variation in how pain is assessed, and how best to interpret findings ^9^. Similarly, the measurement of the impact of pain also varies. Although the National Institutes of Health (NIH) have defined high impact chronic pain as: “associated with substantial restriction of participation in work, social and self-care activities for six months or more ^10^,” there are other ways pain can impact daily living including key psychological aspects (for example, if pain is seen as something to worry about or be scared of), disruption to sleep and associated disability. Measures need to be appropriate for the different stages of the lifespan and cultural context studied. An initial step is to assess what has been collected and to identify where there may be gaps in existing datasets.

Data from existing longitudinal studies, particularly birth cohorts, may include assessments of pain, pain impact as well as biological, psychological and social factors that could be studied to better understand the multifactorial aetiology of pain as well as pain mechanisms. Large population-based cohort studies are pivotal as participants have mostly not experienced the health outcomes of interest at the point of recruitment (depending on the age of participants) and are typically more representative of the general population than clinical pain populations. The Avon Longitudinal Study of Parents and Children (ALSPAC) is such a study, providing a core resource for researchers, including those who are interested in pain. Thus far, a plethora of data has been collected through successive waves of assessments since the birth of the index child participants (Generation 1, G1) throughout childhood, adolescence and into their early adult years. Data have also been collected from their mothers and fathers/the mothers’ partners (Generation 0, G0) and more recently, from their partners and own children (‘The children of the children’ or Generation 2, G2). However, the availability of data on pain can vary over waves of data collection, and there is a need to identify and report what variables exist that could be used to explore pain in this cohort. The overarching aims of this data note were to develop operational definitions for pain categories, collect all pain and pain related variables from G0 and G1 in the ALSPAC cohort and to organise them into pain categories.

## Methods

The ALSPAC cohort invited all pregnant women residing in Bristol and the surrounding area with expected dates of delivery between 1st April 1991 and 31st December 1992 to take part. Initially, 14,541 pregnancies were enrolled out of the eligible 20,248 pregnancies ^11, 12^, resulting in 14,676 foetuses and 14,062 live births. There were 13,988 infants alive at 1 year of age. When the oldest child participants were approximately 7 years of age, there was a booster recruitment campaign, during which eligible children born between April 1991 and December 1992, who had not joined the study during the original recruitment campaign, were invited to join. The sample size for analyses using data from when child participants were 7 years of age is 15,447 pregnancies, resulting in 15,658 foetuses. Of these 14,901 children were alive at 1 year of age. There have been subsequent recruitment drives ^13^. These child participants are also referred to as G1 participants. Data have been collected on all aspects of health and development, including genetics, lifestyle, behaviour, education and work from G1 participants. Multiple methods of data collection have been used including questionnaires (on paper then online in later years), biological assays and direct measurement at face-to-face clinics.

Initially, 14,203 unique mothers were enrolled ^12^. After the booster recruitment campaign, 14,833 unique mothers were enrolled ^14^. Mothers of G1 participants have been completing questionnaires since being recruited and have also attended face-to-face clinics from 17–18 years postnatal ^12^. Partners of the mothers of G1 participants have been involved indirectly or opportunistically (i.e., via the mothers or asked to take part if bringing a G1 participant to a face-to-face clinic) since 1991–1992 but were invited to formally enroll into ALSPAC in 2010. Most of them are the fathers of G1 participants. Data have been collected over 21 questionnaires and 2 face-to-face clinics from 12,113 partners. Of these, 3,807 have been enrolled ^15^. Mothers of child participants (G1) and their partners are also known as G0 participants. We have chosen to focus of G0 and G1 participants as G2 participants are still very young and there has been only very recent involvement of G1 partners.

Online data have been collected and managed using REDCap (Research Electronic Data Capture), hosted at the University of Bristol. REDCap is a secure, web-based software platform designed to support data capture for research studies ^16^. Please note that the study website contains details of all the data that is available through a fully searchable data dictionary and variable search tool: https://www.bristol.ac.uk/alspac/researchers/our-data/. Ethical approval for this study was given by the ALSPAC Ethics and Law Committee (ALEC; IRB00003312) and the NHS Local Research Ethics Committees: Bristol & Weston Health Authority: E1808 Children of the Nineties: Avon Longitudinal Study of Pregnancy and Childhood (ALSPAC) 28/11/1989); Southmead Health Authority: 49/89 Children of the Nineties – "ALSPAC", 05/04/1990); Frenchay Health Authority: 90/8 Children of the Nineties, 28/06/1990. Informed written consent for the use of data collected through questionnaires and clinics was provided by participants following the recommendations of the ALSPAC Ethics and Law Committee at the time. Study participants have the right to withdraw their consent for elements of the study or from the study entirely at any time.

### Identification of pain and pain related variables and operational definitions of pain categories

Our initial step involved searching the ALSPAC data catalogue for key words linked to pain and extracting all relevant variables. Key words searched for included ‘pain’, ‘ache’, ‘sore’, ‘chronic’, specific pain conditions (e.g. migraines, arthritis, rheumatism), pain relief (paracetamol, aspirin, ibuprofen, codeine/anadin, anaesthetics/epidurals during labour, and other), physiotherapy and labour pain. The search identified some variables that were not pain- related, such as variables relating to ‘paint’. Once all relevant pain variables were collated, authors EF, JD and JAD independently developed clinically and conceptually relevant constructs, or pain categories. They then allocated variables to the most suitable pain categories. Multiple consensus meetings were then held to reach agreement on the final list of pain categories and the categorisation of all relevant pain variables. During this iterative process, some pain categories were dropped, renamed and added; there was adjudication by 2 or more researchers when there was disagreement on where to put relevant variables. AJ reviewed the allocation of pain variables and performed final adjudication. Agreed pain categories, which were represented by available variables, are described below with examples.

***Pain characteristics***

•   Pain frequency – how often pain was experienced over a given period, e.g., number of times respondent has had pain in their wisdom teeth

•   Pain duration – how long pain was experienced for over a given period, e.g., young person has had any aches/pain that lasted for a day/longer in the past month

•   Pain magnitude/scale/intensity - assessment of pain severity, e.g., severity of period-associated pain

•   Pain site – locations of pain experienced, e.g., participant had chest pain in October

•   Pain-related affect – any emotional reaction, feeling or consequence related to being in pain, e.g., child thinks headaches are brought on by being worried

***Extended pain characteristics and causes***

•   Presence of pain – any pain that was experienced within specific timeframes, e.g., participant had sore throat in Oct 2020

•   Circumstance of pain – the context in which pain was experienced, e.g., young person got stomach aches at college/university/work/public places

•   Associated pain - any pain associated with movement or event, e.g., young person gets pain in their face when they move their jaw

•   Associated pain-related events – events that were triggered by or associated with the pain, e.g., child is over-sensitive to pain: hearing/hyperacusis

•   Perceived causes of pain – causes of pain that were not confirmed by any formal assessment, e.g., child thinks headaches are brought on by noise

•   Implied pain caused by trauma – pain not always explicitly described but implicated as a result of bodily trauma, e.g., young person still experiences problems with nearby joints as a result of previous fracture

•   Specific condition – any syndrome, disease or disorder relating to pain, e.g., Mother ever had migraine

***Treatment for pain***

•   Pain treatment – treatment given to manage pain. It could be self-administered or given by a healthcare professional, e.g., young person had physiotherapy as a result of fracture

•   Specialist review – when attention from a healthcare professional was sought to assess and treat pain, e.g., Doctor contacted about period-associated severe cramps

***Pain interference***

•   Affective pain interference – any emotion-related pain distress or interference that may have impacted daily life, e.g., afraid to move due to pain

•   Pain-related physical interference – how pain may have impacted physical movement, functioning or level of independence, e.g., young person still experiences difficulty using the area as a result of fracture

•   Pain-related social interference – how pain may have affected relationships and different aspects of social well-being, e.g., ability to fit in with friends and progress at school (NB. Some variables include how to deal with problems and progress in school/college/work, which more broadly is social)

•   Troublesome/Bothersome pain ratings- pain or discomfort that affected a part of the body that was difficult to ignore, e.g., young person’s neck pain has been troublesome in the past month

•   Pain-related sleep interference, e.g., reason for sleep difficulties, with pain as a possible cause

***Pain related to specific events***

•   Pregnancy, birth, and postpartum related pain - any pain that was experienced and sometimes treated during pregnancy and the postpartum period of 2 months post birth, e.g., pain level in labour

•   Pain experienced during COVID-19 – data collected during the COVID-19 pandemic, e.g., abdominal pain in May: COVID3

All variables were organised into worksheets in a single Microsoft Excel spreadsheet from G1 self-administered questionnaires, G1 Focus clinics, and for mothers and partners. There is also an index worksheet, which focusses on pain sites and strategies to treat pain. The first 5 puberty questionnaires were completed by either parent or child and thus were excluded from the child-completed section, given the uncertainty in reporter. However, any repeated assessments of pain, irrespective of whether they were self- or proxy- reported feature in the repeated measures section below, including from these puberty questionnaires. There is also a worksheet documenting pain variables from G1 participants’ partners from some of the COVID waves but they are not summarised in this data note. These worksheets are now separate spreadsheets that can have been shared publicly on Open Science Framework; more details can be found in the Extended data section. Each questionnaire and corresponding dataset have been given file names to index them, providing a way to find datasets of interest. For example, G1 child-completed questionnaires typically start with letters that may indicate age or content (e.g. YP = young person, PUB = puberty) and are in chronological order. These were named by the ALSPAC team, not the researchers involved in this data note. More details on file names can be found on the ALSPAC website under questionnaires https://www.bristol.ac.uk/alspac/researchers/our-data/questionnaires/.

## Results

### Pain variables in questionnaires from G1 participants

Pain variables in ALSPAC have been collected on G1 participants primarily via self-report questionnaires (referred to by ALSPAC as child-completed) or completed by a parent or other adult such as a teacher about the child (referred to by ALSPAC as child-based). This section focusses on pain variables collected directly from G1 participants via child-completed questionnaires, including the puberty questionnaires. From the age of 65 months, there have been multiple waves of data collection during which child participants completed questionnaires on their own or with some assistance from an adult (specified as a ‘grown-up’ in the questionnaires). In Table 1, we list the categories of pain and the number of pain variables available in them. Most variables about pain relate to pain site, pain frequency, pain presence and pain magnitude. Many pain assessments were also collected during the COVID-19 data waves (2020 to 2021). There are comparatively fewer variables in categories such as pain treatments, specialist review, pain-related physical interference and pain-related social interference, highlighting an important gap in data availability in these categories.

### Pain variables from face-to-face clinics from G1 participants

G1 participants completed pain-related questions at 4 ‘Focus’ clinics, at ages 10, 15 years and 6 months, 17 years and 24 years (respectively named Focus@10 (F10), TeenFocus 3 (TF3), TeenFocus4 (TF4), and Focus@24 (F24)). These clinics were open to all members of ALSPAC; a range of data were collected in these clinics, including anthropometry and measures of vision and hearing. Further information can be found here: https://www.bristol.ac.uk/media-library/sites/alspac/documents/researchers/clinics/focusclinicsessions.pdf. In the third column of Table 1, we list the number of pain variables in each pain category from the Focus clinics. Most pain variables relate to the site of pain, pain duration, associated pain-related events and pain-related affect. There were comparatively fewer variables in other categories, with none in perceived causes of pain, pregnancy, birth and postpartum related pain, circumstance of pain and COVID-19.

**Table 1.** Pain and pain-related variables on G1 in ALSPAC, child completed questionnaires and Focus clinics.

Category	Counts of variables	
	Child-completed	Focus clinics
Pain frequency	57	5
Pain duration	5	23
Pain magnitude/scale/intensity	38	14
Pain site	256	114
Associated pain-related events	24	30
Pain treatment	9	3
Specialist review	8	3
Pain-related affect	1	21
Affective pain interference	0	1
Pain-related physical interference	10	9
Pain-related social interference	10	9
Troublesome/bothersome pain ratings	19	4
Pain-related sleep interference	0	1
Perceived causes of pain	13	0
Pregnancy, birth and postpartum related pain	0	0
Presence of pain	106	15
Specific condition	18	6
Associated pain	14	2
Implied pain caused by trauma	0	12
Circumstance of pain	2	0
COVID 19	168	0

G1 is an abbreviation for ‘Generation 1’, i.e. the child participants recruited into the study. ALSPAC is an abbreviation for Avon Longitudinal Study of Parents and Children. Child-completed refers to questionnaires that G1 participants completed themselves. Focus clinics were in-house data collection waves during which many types of data were collected including via questionnaire and physical measurement.

### Repeated measures

**Figure 1.**
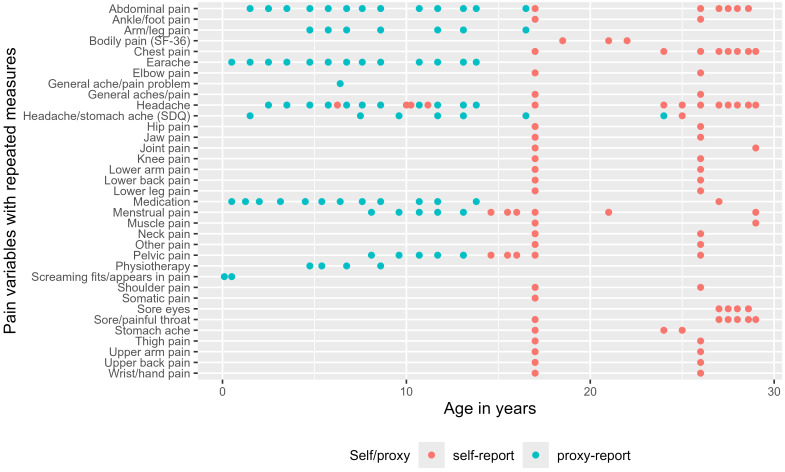
Timeline for repeated measures relating to pain in G1 ALSPAC participants. Pain or treatment of pain has been repeatedly measured over the span of 30 years follow up in ALSPAC. This Figure provides visual representation of when pain data were collected and whether the data were collected via self-reports or proxy-reports for G1 participants. Only pain measures that were repeatedly assessed are shown here.

This section focusses on pain variables that were collected via self-report questionnaires and from Focus clinics from when G1 participants were aged 65 months until December 2021, when they were in their late 20s. Pain variables were also collected in proxy-report (child-based) questionnaires from when G1 participants were 4 weeks old; many of these variables were collected repeatedly. Figure 1 is a timeline that shows during which wave and age repeated measurements of pain were collected. Maternal proxy-reports were more common when G1 participants were in childhood and adolescence. Self-report was almost solely adopted from adolescence onwards in both Focus clinics and questionnaires. Repeated measurements are predominantly on pain site. There is a density of available data from the data collection waves at ages 17–18 (CCT and TF4 questionnaires), age 26 (YPF questionnaire) and during the COVID-pandemic. We also observe that there are repeated measurements of menstrual and pelvic pain from the puberty questionnaires during adolescence. Figure 1 is based on data from the index spreadsheet; more details can be found in the Extended data section. Please note there are also repeated measures for mothers and partners, as documented in the index spreadsheet, but are not highlighted here.

## G0 Mothers and fathers/mothers’ partners

### Pain variables from G0 mothers

This section focusses on G0 mothers of G1 child participants. In Table 2, we list the number of variables in each pain category. In some categories such as pain site, presence, COVID-19 and pain frequency, over 100 pain-related variables are available. There are comparatively fewer variables in other categories such as pain duration, pain magnitude, associated pain-related events, pain treatment, specialist review, pain-related affect, etc.

**Table 2.** Pain and pain-related variables on G0 mothers in ALSPAC, self-administered questionnaires.

Category	Counts of variables
Pain frequency	123
Pain duration	25
Pain magnitude/scale/intensity	42
Pain site	474
Associated pain-related events	53
Pain treatment	80
Specialist review	17
Pain-related affect	1
Pain-related physical interference	5
Pain-related social interference	5
Perceived causes of pain	1
Pregnancy-related	47
Presence	291
Specific condition	1
Associated pain	4
Implied pain caused by trauma	4
Circumstance of pain	19
COVID 19	197

G0 is an abbreviation for ‘Generation 0’, i.e. the parents of child participants recruited into the study, or occasionally the mothers’ partners. ALSPAC is an abbreviation for Avon Longitudinal Study of Parents and Children.

### Pain variables from G0 partners

Pain-related variables from the G0 partners are from questionnaires. Table 3 is a summary of all the available pain-related variables with the number of variables in each pain category. There are variables in the categories of COVID 19, pain site, presence, pain frequency, pain magnitude and pain treatment. Data were not available in other categories.

**Table 3.** Pain and pain-related variables on G0 partners in ALSPAC, self-administered questionnaires.

Category	Counts of variables
Pain frequency	19
Pain magnitude/scale/intensity	19
Pain site	113
Pain treatment	2
Presence	102
COVID 19	149

G0 is an abbreviation for ‘Generation 0’, i.e. the parents of child participants recruited into the study, or occasionally the mothers’ partners. ALSPAC is an abbreviation for Avon Longitudinal Study of Parents and Children.

## Data considerations

ALSPAC provides a wealth of data for pain research including, for example, into pregnancy and menstrual health. Longitudinal cohort study designs and family trio study designs can be implemented with the existing data from the ALSPAC cohort, linking pain data to, for example, data on education, the built environment and medical records. With data collected from birth through to early adulthood on G1 participants, researchers are well placed to study pain experienced during these earlier stages of the life course. As a multi-generational cohort, maternal and paternal exposures on pain in offspring can also be effectively investigated with ALSPAC data. This dataset is also well suited to studying pain during the COVID 19 pandemic and also mothers who have been followed up for several years since the perinatal period.

ALSPAC, at the time of recruitment, was mostly representative of Bristol and surrounding areas. However, it is noted that compared to those in the former county of Avon and the whole of Great Britain ALSPAC participants are of a slightly higher socioeconomic position and there is a smaller proportion of individuals from an ethnic minority background, limiting the generalisability of findings ^11, 12^. Like other cohort studies, there has inevitably been attrition of participants within the ALSPAC cohort. To give an impression of the extent of the attrition, 4348 G1 participants responded to the Life at 29+ questionnaire. Various methods to handle missingness in the dataset have been used in other studies, such as multiple imputation and full information maximum likelihood ^17, 18^. These more complex methods are not always needed, complete case analyses can produce unbiased estimates, even with missing not at random (MNAR) present ^19^. Considerations on handling missing data should be made for each specific research question considering the links between drop out and both exposures and pain outcomes.

One of the inevitable limitations with any cohort dataset is that not everything of interest will have been measured and in a consistent way (if taken over multiple waves). In addition, over time, concepts that are thought to be relevant change. There are current efforts to address gaps in the availability of data on pain by collecting further data on pain at ages 30 and 31 as part of the CRIISP (Consortium to Research Individual, Interpersonal and Social influences in Pain) project, which is funded with the UK’s Advanced Pain Discovery Platform (APDP). Some of the same questions about pain status, pain intensity and pain impact that were asked at the TF4 data collection clinic, are also being asked at ages 30 and 31. Further questions on psychological and social phenomena that may be associated with pain, for example, psychological aspects of pain, loneliness, social support, discrimination, work and impressions left after participants’ interactions within the healthcare system have also been added. Together this will increase the research potential of ALSPAC for those interested in better understanding pain.

## Disclaimer

Any dissemination of results, communication activity must indicate that it reflects only the authors’ views and that the research council is not responsible for any use that may be made of the information it contains.

### Example

The views expressed are those of the authors and not necessarily those of the Medical Research Council or Versus Arthritis.

## Ethics and consent

Ethical approval for this study was given by the ALSPAC Ethics and Law Committee (ALEC; IRB00003312) and the Local NHS Research Ethics Committees: Bristol & Weston Health Authority: E1808 Children of the Nineties: Avon Longitudinal Study of Pregnancy and Childhood (ALSPAC) 28/11/1989); Southmead Health Authority: 49/89 Children of the Nineties – "ALSPAC", 05/04/1990); Frenchay Health Authority: 90/8 Children of the Nineties, 28/06/1990. Informed written consent for the use of data collected through questionnaires and clinics was provided by participants following the recommendations of the ALSPAC Ethics and Law Committee at the time. Study participants have the right to withdraw their consent for elements of the study or from the study entirely at any time

## Data Availability

ALSPAC data access is through a system of managed open access. The steps below highlight how to apply for access to the data included in this paper and all other ALSPAC data. Note that variable names are included in the tables within this paper. Please read the ALSPAC access policy ( http://www.bristol.ac.uk/media-library/sites/alspac/documents/researchers/data-access/ALSPAC_Access_Policy.pdf) which describes the process of accessing the data and biological samples in detail, and outlines the costs associated with doing so. 1. You may also find it useful to browse our fully searchable research proposals database ( https://proposals.epi.bristol.ac.uk/), which lists all research projects that have been approved since April 2011. 2. Please submit your research proposal ( https://proposals.epi.bristol.ac.uk/) for consideration by the ALSPAC Executive Committee using the online process. You will receive a response within 10 working days to advise you whether your proposal has been approved. If you have any questions about accessing data, please email: alspac-data@bristol.ac.uk (data) or bbl-info@bristol.ac.uk (samples). The ALSPAC data management plan ( http://www.bristol.ac.uk/media-library/sites/alspac/documents/researchers/data-access/alspac-data-management-plan.pdf) describes in detail the policy regarding data sharing, which is through a system of managed open access. Open Science Framework: Data note for pain data in the Avon Longitudinal Study of Parents and Children. https://doi.org/10.17605/OSF.IO/ZPSG9 ^20^ This project contains the following extended data: Final Child_pain_ALSPAC.xlsx Final Child Clinic_pain_ALSPAC.xlsx Final Mother_pain_ALSPAC.xlsx Final Partners_pain_ALSPAC.xlsx Final YP partner_pain_ALSPAC.xlsx Index_pain_ALSPAC.xlsx This data note predominantly focuses on the spreadsheets called ‘Final Child_pain_ALSPAC’, ‘Final Mother_pain_ALSPAC’ and ‘Final Partners_pain_ALSPAC’ and ‘Final Child Clinic_pain_ALSPAC’. We also used the columns ‘Child-completed’, ‘Child-based’ and ‘Child’ from the ‘Index_pain_ALSPAC’ spreadsheet to create Figure 1 for repeated measures. We have flagged in the data note where we have not described or summarised the data in some detail. These are: columns called ‘Mother’, ‘Partner’, ‘Young person’s partner’ in the ‘Index_pain_ALSPAC’ spreadsheet and ‘Final YP partner_pain_ALSPAC’ spreadsheet. Data are available under the terms of the Creative Commons Attribution 4.0 International Public License (CC-BY Attribution 4.0 International)
