# Supplementary material for: A summary of pain and pain-related variables in the Avon Longitudinal Study of Parents and Children
Source: Wellcome Open Res. 2024 Sep 12;9:521. [Version 1] doi: 10.12688/wellcomeopenres.22815.1 (PMC11462121; doi:10.12688/wellcomeopenres.22815.1)
Supplement: Peer review history

# Reviewer report: Reviewer response for version 1

**DOI:** 10.21956/wellcomeopenres.25121.r101714

**Published:** 2024-10-08

**Authors:** Juliana Choueiry

**Affiliations:** 1 University of Ottawa, Ottawa, Ontario, Canada

Thank you for the opportunity to review your article. I appreciate the hard work and time put into this to improve the operational definitions of pain-related variables and categories. The pain categories are listed clearly and can be beneficial for future use in practice. The use of these categories to assess pain can help clinicians ensure a comprehensive assessment of pain. It would be interesting to assess the comprehensiveness of existing pain assessment scales using these categories and identify what facets of pain are not captured within the standardized scales. 

**Here are a few suggestions to improve this article: **

- Recommend elaborating on the description and distinction of high vs low impact of pain earlier on in the background when first introduced. What does a low impact look like? How do we determine what is high or low and why is it important to distinguish these? 
- You define the high impact of chronic pain but what about the impact of acute pain? Maybe worth mentioning briefly about impact of acute pain. I would also move this information to the previous section when you first introduce the impact of pain to facilitate reading and comprehension of this concept. 
- Recommended elaborating on variations in pain assessment and interpretations of findings. Can be a short one-liner providing examples of this variation. 
- Recommend elaborating and clarifying the data collected. When introducing this, you note "a plethora of data has been collected through successive waves of assessments". To clarify, is this pain-related data or all data from generalized assessments? This is not very clear to me when reading into the methods section. 
- When introducing questionnaires, what type of questionnaires were used? Is this to collect demographic information or other? I would add more details here. 
- I found it a bit confusing to remember who G1 participants were vs other and had to refer back to the previous section to recall, which interrupted the flow of reading through this.
- I assume that the Focus clinics are the face-to-face clinics previously mentioned. For clarity, it may be beneficial to introduce the name of "Focus" clinics earlier when introducing face-to-face clinics. What are focus clinics? 

Thank you!

Are sufficient details of methods and materials provided to allow replication by others?

Yes

Is the rationale for creating the dataset(s) clearly described?

Yes

Are the datasets clearly presented in a useable and accessible format?

Yes

Are the protocols appropriate and is the work technically sound?

Yes

Reviewer Expertise:

Pediatric pain, family-centred care, children with cognitive impairments

I confirm that I have read this submission and believe that I have an appropriate level of expertise to confirm that it is of an acceptable scientific standard.

# Reviewer report: Reviewer response for version 1

**DOI:** 10.21956/wellcomeopenres.25121.r99239

**Published:** 2024-09-30

**Authors:** Annalisa Hauck

**Affiliations:** 1 Paediatric Neuroimaging Group, University of Oxford, Oxford, England, UK

The data note summarises the pain and pain-related variables availabe in the Avon Longitudinal Study of Parents and Children across two generations of cohorts and over 30 years. This data note is clear and concise. 

 It provides a valuable overview to help researchers select the most appropriate variables  and to better understand the data available within the dataset. As the authors point out, the dataset was not originally created to specifically collect pain-related data. This limitation is already adressed by the authors, who provide a transparent overview of limitations, as well as of the steps being taken to fill the gaps in the data (e.g., through ongoing studies) and to take into account missing data. Overall, the dataset provides a wealth of data on pain and pain-related variables, as well as on genetics, health measures and further variables.

**I have the following minor suggestions for clarification and context:**

- General note: I would add a mention of other datasets which are being deleloped to study pain in childhood  (e.g., https://www.ncbi.nlm.nih.gov/pmc/articles/PMC7309240/) and how the ALSPAC dataset differs from it and provides further valuable data.
- I suggest adding a CONSORT flow diagram to provide a brief overview of paragraphs 1-2 of the Methods section, which refer to the number of participants (pages 7-8)
- How did you perform the key word search on the paper data? Did you first transfer all data to a digital form? (page 7)
  - **`**Multiple methods of data collection have been used including questionnaires (on paper then online in later years), biological assays and direct measurement at face-to-face clinics. **'** 
- How did you select the **`**conceptually relevant constructs **'** (page 8)? Was the selection based on other studies on pain or primarily on internal consensus based on the available data? 
- When referring to the multiple consensus meetings held, do I understand correctly that these were held among the three researchers named EF, JD and JAD without further stakeholders? (page 8). If so, please specify.
- Self-report is mainly used from adolescence onwards. Were there specific criteria to decide when to opt for self-report and when to opt for proxy-report (or can these criteria be looked up in a different document which could be linked for reference)?

Thank you to the authors for developing, curating and sharing this valuable dataset.

Are sufficient details of methods and materials provided to allow replication by others?

Yes

Is the rationale for creating the dataset(s) clearly described?

Yes

Are the datasets clearly presented in a useable and accessible format?

Yes

Are the protocols appropriate and is the work technically sound?

Yes

Reviewer Expertise:

Neuroimaging, pain assessment and treatment in early of life.

I confirm that I have read this submission and believe that I have an appropriate level of expertise to confirm that it is of an acceptable scientific standard.

## References

[1] : Developing a pediatric pain data repository. *JAMIA Open* .2020;3(1) : 10.1093/jamiaopen/ooz062 31-36 10.1093/jamiaopen/ooz062 32607485 PMC7309240
